# First Miocene rodent from Lebanon provides the 'missing link' between Asian and African gundis (Rodentia: Ctenodactylidae)

**DOI:** 10.1038/srep12871

**Published:** 2015-08-07

**Authors:** Raquel López-Antoñanzas, Fabien Knoll, Sibelle Maksoud, Dany Azar

**Affiliations:** 1 School of Earth Sciences, University of Bristol, Bristol, United Kingdom; 2 Departamento de Paleobiología, Museo Nacional de Ciencias Naturales-CSIC, Madrid, Spain; 3 School of Earth, Atmospheric and Environmental Sciences, University of Manchester, Manchester, United Kingdom; 4 UMR 6538 Domaine Océanique, Université de Bretagne Occidentale, Brest, France; 5 Natural Sciences Department, Faculty of Sciences II, Lebanese University, Fanar, Lebanon

## Abstract

Ctenodactylinae (gundis) is a clade of rodents that experienced, in Miocene time, their greatest diversification and widest distribution. They expanded from the Far East, their area of origin, to Africa, which they entered from what would become the Arabian Peninsula. Questions concerning the origin of African Ctenodactylinae persist essentially because of a poor fossil record from the Miocene of Afro-Arabia. However, recent excavations in the Late Miocene of Lebanon have yielded a key taxon for our understanding of these issues. *Proafricanomys libanensis* nov. gen. nov. sp. shares a variety of dental characters with both the most primitive and derived members of the subfamily. A cladistic analysis demonstrates that this species is the sister taxon to a clade encompassing all but one of the African ctenodactylines, plus a southern European species of obvious African extraction. As such, *Proafricanomys* provides the 'missing link' between the Asian and African gundis.

The Ctenodactylinae is a subgroup of the Ctenodactylidae (Ctenohystrica) that likely appeared around the Oligocene-Miocene boundary. These unique animals have since experienced a remarkable evolution involving both a shift in habitats (from moist to arid) and distribution (from Asia to Africa). Our understanding of the phylogenetic relationships within the clade has been recently much improved^1^. Further progresses will intimately rely on efforts to increase our knowledge of the fossil record of the group, which remains sporadic. Special focus on the Miocene and the Arabian Peninsula is critical in this respect as this is when and where the ctenodactylines migrated from the Eurasian plate. These rodents are currently known from a small number of Miocene sites on the Arabian Plate. The fossil localities of the Rotem Basin (MN 3-MN 4 transition equivalent, Early Miocene, ~18 Ma) in Israel, which have yielded an undescribed species of *Sayimys*^2^, are situated to the West of the Dead Sea Transform and, therefore, on the African plate. The slightly younger localities of the As-Sarrar region in Saudi Arabia (MN 5 equivalent, Early Miocene, ~16 Ma) have yielded *Sayimys assarrarensis*^3^,^4^. *Sayimys intermedius* has been recorded from the site of Tayma (MN 5 equivalent, Middle Miocene, ~15 Ma), Saudi Arabia^3^,^4^. The type locality of the latter species is Al Jadidah (MN 6 equivalent, Middle Miocene age, ~13 Ma), Saudi Arabia^3^,^4^,^5^. If this record may appear poor, it should be stressed that to date no Arabian Late Miocene site had yielded any ctenodactyline remains. The only localities of appropriate age, those of the Al Gharbia region, United Arab Emirates (MN 13 equivalent, Late Miocene, ~7 Ma), have produced rodents^6^,^7^,^8^,^9^, but not any ctenodactylines. Given this background, we conducted extensive field prospecting in May-June of 2013 in the continental Late Miocene of Lebanon. Fossil mammals of this age were first reported in Lebanon more than half a century earlier^10^, but only the remains of the most common component (horses) were described in some detail^11^.

Our labour in Lebanon resulted in the unforeseen discovery of a variety of new fossil sites immediately to the East of the Dead Sea Transform (Yammouneh Fault). One of the localities has yielded vertebrate remains, mainly fishes, turtles, and crocodiles, but also micromammals, including the first Late Miocene ctenodactyline from the Arabian Plate. This site is situated close to the spring of Aïn-el-Daouk, immediately North-West of the town of Zahleh (Bekaa Valley, central Lebanon). It is a road cut whose stratigraphic sequence is presented in Fig. 1. A bone fragment (which proved, after preparation, to be a large piece of turtle shell) protruding from a brown fossiliferous silty layer (numbered 3 in Fig. 1) prompted us to extract about 500 kg of rock and screenwash it in the field.

Dubertret and Vautrin^12^ first attributed these deposits to the 'Pontian' (Messinian), but without paleontological support. The work of Haj-Chahine^13^ seemed to confirm this age on the basis of the vertebrate content. However, the new taxon of ctenodactyline described below, together with the presence of *Progonomys* sp. at the same level, supports a correlation with either the European MN 10 or MN 11 (~9 Ma) (see discussion below). Layers yielding vertebrates are also exposed near Kafraya, some 25 km South-West of Aïn-el-Daouk (Zahleh)^10^,^11^,^14^. As a matter of fact, during Late Miocene time a considerable part of the Bekaa Valley was covered by a lake. Walley^15^ informally termed the lacustrine deposits in Zahleh as the 'Zahleh Formation'.

The aim of the present work is to describe the ctenodactyline teeth uncovered at this locality and analyse the significance of this novel taxon.

## Results

### Systematic paleontology

Rodentia Bowdich, 1821

Ctenohystrica Huchon, Catzeflis et Douzery, 2000

Ctenodactylidae Gervais, 1853

*Proafricanomys* nov. gen.

**Figure 2:**
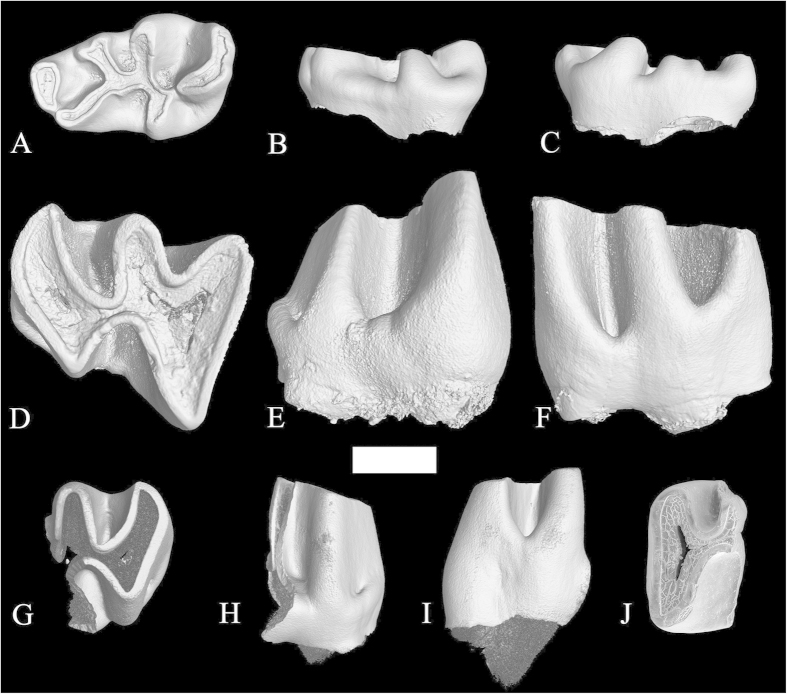
Lower cheek teeth of *Proafricanomys libanensis* nov. gen. nov. sp. **(A–C)** Zahleh 13, left dp4. **(A)** occlusal view; **(B)** labial view; **(C)** lingual view. **(D–F)** Zahleh 28, right m3. **(D)** occlusal view; **(E)** labial view; **(F)** lingual view. **(G–I)** Zahleh 10, posterior fragment of left m1. **(G)** occlusal view; **(H)** labial view; **(I)** lingual view. **(J)** Zahleh 3, anterior fragment of left m1, occlusal view. 3D rendering from X-ray microtomography (μCT scan). Scale bar equals 1 mm.

**Figure 3:**
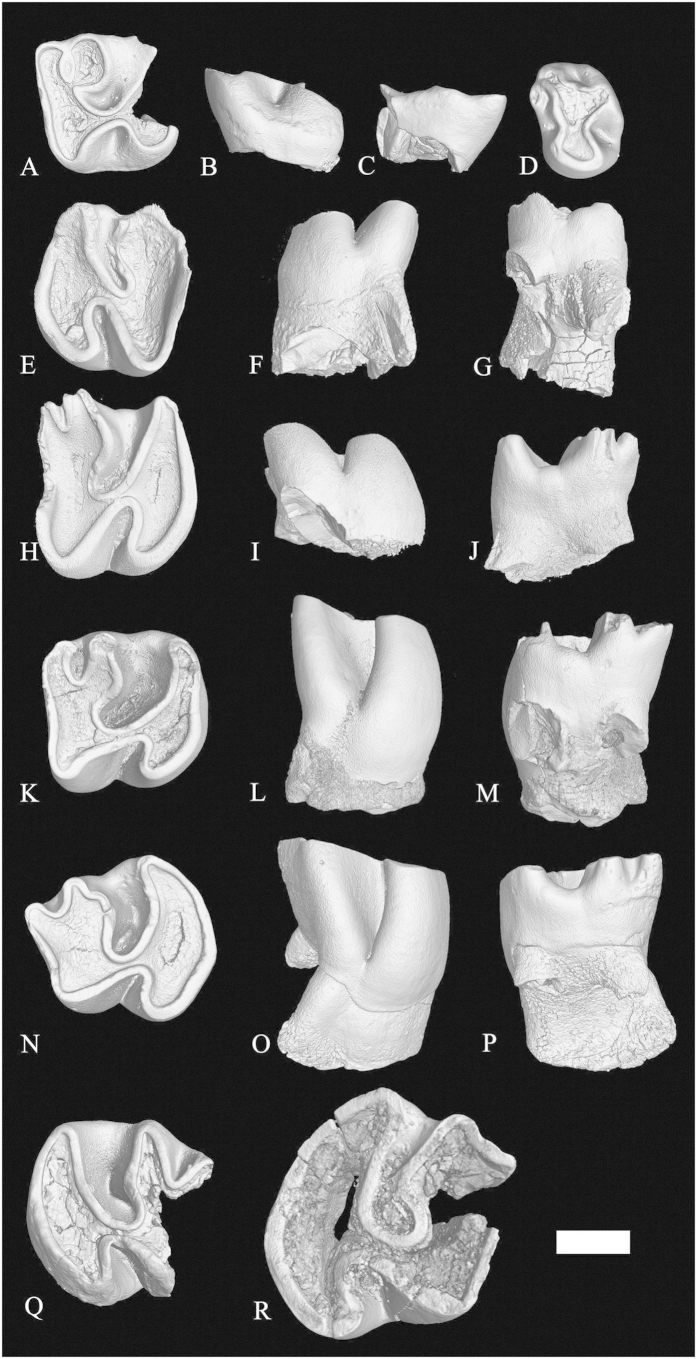
Upper cheek teeth of *Proafricanomys libanensis* nov. gen. nov. sp. **(A–C)** Zahleh 7, right DP4. **(A)** occlusal view; **(B)** lingual view; **(C)** labial view. **(D)** Zahleh 6, left P4, occlusal view. **(E–G)** Zahleh 26, left M1. **(E)** occlusal view; **(F)** lingual view; **(G)** labial view. **(H–J)** Zahleh 25, right M1. **(H)** occlusal view; **(I)** lingual view; **(J)** labial view. **(K–M)** Zahleh 47, right M1. **(K)** occlusal view; **(L)** lingual view; **(M)** labial view. **(N–P)** Zahleh 46, right M2. **(N)** occlusal view; **(O)** lingual view; **(P)** labial view. **(Q)** Zahleh 23, left M2, occlusal view. **(R)** Zahleh 12, left M2, occlusal view. 3D rendering from X-ray microtomography (μCT scan). Scale bar equals 1 mm.

**Figure 4:**
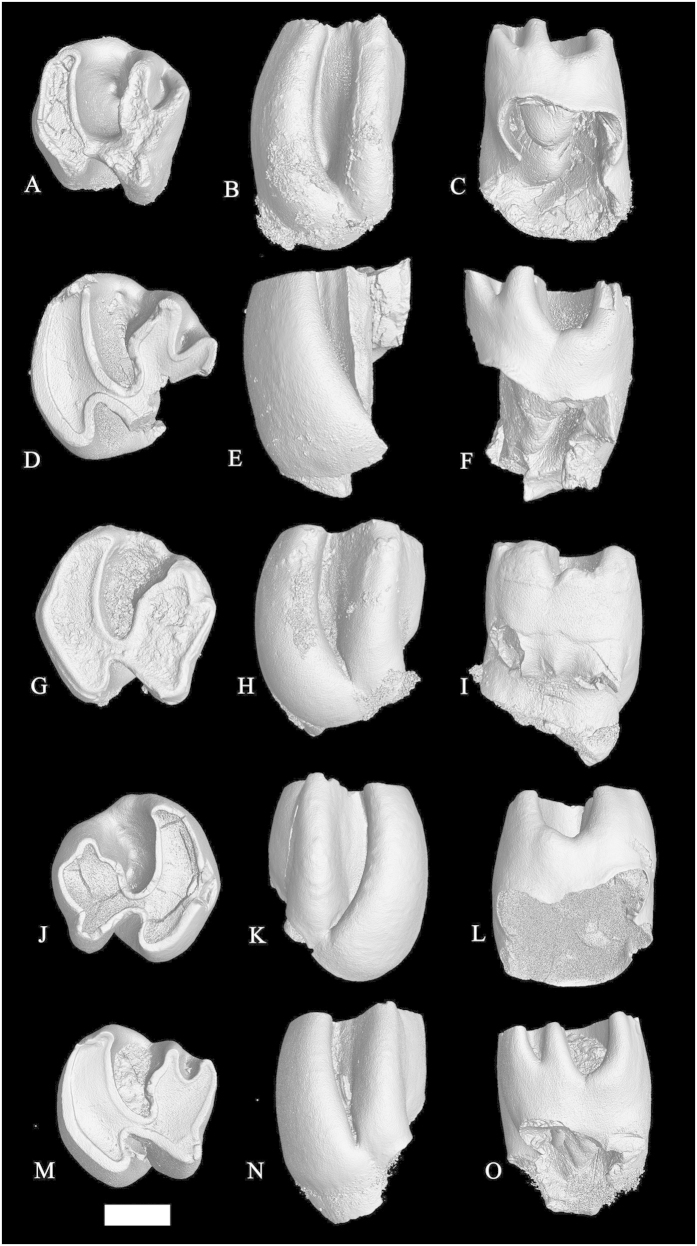
Upper cheek teeth of *Proafricanomys libanensis* nov. gen. nov. sp. **(A–C)** Zahleh 24, left M2. **(A)** occlusal view; **(B)** lingual view; **(C)** labial view. **(D–F)** Zahleh 9, left M2. **(D)** occlusal view; **(E)** lingual view; **(F)** labial view. **(G–I)** Zahleh 27, left M2-3. **(G)** occlusal view; **(H)** lingual view; **(I)** labial view. **(J–L)** Zahleh 22, left M2-3. **(J)** occlusal view; **(K)** lingual view; **(L)** labial view. **(M–O)** Zahleh 11, right M3. **(M)** occlusal view; **(N)** lingual view; **(O)** labial view. 3D rendering from X-ray microtomography (μCT scan). Scale bar equals 1 mm.

*Proafricanomys libanensis* nov. sp. (Figs 2, 3, 4)

#### Etymology

The generic epithet is composed by the prefix *pro-* and *Africanomys*, indicating that this taxon is a possible forerunner of the latter genus. The specific name means 'from Lebanon'.

#### Holotype

left dp4 (Zahleh 13) (Fig. 2A–C and Supplementary Figure S1), This and the paratype specimens are provisionally housed in the National Museum of Natural Sciences-CSIC in Madrid. They will be stored in the Lebanese University in Fanar upon completion of their study.

#### Paratypes

left m1-2 anterior and posterior fragments (Zahleh 3, Zahleh 10, Fig. 2G–J); right m3 (Zahleh 28, Fig. 2D–F); right DP4 (Zahleh 7, Fig. 3A–C); left P4 (Zahleh 6, Fig. 3D and Supplementary Figure S2); left M1 (Zahleh 26, Fig. 3E–G); right M1 (Zahleh 25, Zahleh 47, Fig. 3H–M); right M2 (Zahleh 46, Fig. 3N–P), left M2 (Zahleh 23, Zahleh 12, Zahleh 24, Zahleh 9, Figs 3Q–R and 4A–F); left M2-3 (Zahleh 27, Zahleh 22, Fig. 4G–L); right M3 (Zahleh 11, Fig. 4M–O).

#### Type Locality

Zahleh (Lebanon)

**Age.** Late Miocene (Tortonian)

#### Diagnosis

Ctenodactylinae with unilateraly semi-hypsodont cheek teeth. dp4 without metalophulid I but with well-developed anterolingually directed metalophulid II; protoconid very anteriorly located, nearly in contact with the isolated large anteroconid, and having a very elongated posterior arm; metaconid situated as in *Sayimys* spp. but joined with the metalophulid II instead of with the metalophulid I; lack of connection between the hypoconid and the posterolophid; lower molars with mesoflexid shorter but deeper than the metaflexid, with a well-developed posterolabial ledge, and lacking a constriction in the posterolophid; P4 with cusp-like anteroloph and high posteroloph with both labial and lingual extensions; upper molars in which anteroloph and protoloph tend to fuse after little wear; M3 posteriorly reduced.

#### Differential Diagnosis

Differing from *Prosayimys flynni*, *Sayimys assarrarensis, Sayimys obliquidens, Sayimys giganteus, Sayimys intermedius, Sardomys dawnsonae, Pireddamys rayi*, and *Metasayimys curvidens*, in lacking the metalophulid I on the dp4, in having the paraflexus on the upper molars obliterated very early in wear and the posterior side of the M3 reduced; distinct from *Sardomys*, *Pellegrinia*, and *Metasayimys* in lacking cement filling the valleys on the lower molars. Differing from *Sayimys intermedius* and *Sayimys sivalensis* in having metalophulid II on the dp4 and the mesoflexid shorter than the metaflexid on the lower molars. Differing from *Sayimys baskini* and *Sayimys sivalensis* in lacking metalophulid I on the dp4. Differing from *Africanomys* spp. in having the metaconid on the dp4 more posteriorly located, and from all but *Africanomys* cf. *solignaci* in having a distinct anteroconid and the metalophulid II anterolingually directed instead of anteriorly-oriented on the dp4. Distinct from *Irhoudia, Pellegrinia*, *Felovia*, *Massoutiera*, and *Ctenodactylus*, in being much less hypsodont, and from *Irhoudia, Pellegrinia*, and all living ctenodactylines in having anteroconid on the dp4 and a distinct metaflexus on the upper molars.

#### Description

dp4 (Zahleh 13)–The pattern of the dp4 is lophate. Its occlusal outline is subrectangular, elongate and narrow (Fig. 2A–C, Supplementary Information S1). The broadened anteroconid is isolated. This specimen is of particular interest because it has a well-developed anteroconid and a metalophulid II but lacks the metalophulid I. The posterior position of the metalophulid II, which connects near the end of the posterior arm of the protoconid, determines the V-shape of the mesoflexid, which is shorter than the metaflexid. The absence of metalophulid I makes the anteroflexid (anterior valley) wide. The metaconid seems to be composed of two small fused cuspids and connects to the metalophulid II. The protoconid is smaller than the hypoconid. The former is very anteriorly located, nearly in contact with the anteroconid and has the posterior arm very elongated. The hypoconid is isolated from the posterolophid by a shallow incision. The ledge on the posterolabial side is weak. This tooth has no preserved roots.

Lower molars–Unfortunately, two of the three lower molars available are broken.

m1-2–One specimen (Zahleh 3, Fig. 2J) has only its anterior side preserved, whereas only the posterior side is conserved in the other one (Zahleh 10, Fig. 2G–I). The m1-2 of *Proafricanomys libanensis* have a mesoflexid that is shorter than the metaflexid, a roughly transverse hypolophid, a long and unconstricted posterolophid, and a distinct low posterolabial cingulum. These teeth have no preserved roots.

m3–The single complete specimen is a right m3 (Fig. 2D–F). The outline of the occlusal surface of this tooth is posteriorly curved. The mesoflexid is much shorter and deeper than the metaflexid. The mesoflexid is shallower than the hypoflexid. The hypolophid is more oblique than in the m1-2 and is not exactly opposite the hypoflexid. The protoconid is larger and extends much more labially than the hypoconid. The posterolophid does not constrict before reaching the triangular wear surface of the hypoconid. This tooth shows a low and well-developed cingulum on its postero-labial side. It is larger than the m1-2. It has no preserved roots.

DP4–This tooth is smaller, much less high-crowned, and has the hypoflexus wider and the hypostria much shallower than on the upper molars. The single available DP4 is anteriorly broken (Zahleh 7, Fig. 3A–C) and it is not possible to see if the anteroloph is fused with the protoloph. The posteroloph is located near the metacone and a short (comma-shaped) metaloph connects distally the two structures. The protocone and hypocone are equally developed and connected by a short and straight endoloph. The roots of this tooth are not preserved.

P4–The occlusal outline of the P4 is nearly oval (Fig. 3D, Supplementary Figure S2). The protocone is better developed than the paracone. The anteroloph is cusp-like and it is nearly in contact with the paracone on the anterolabial corner of the tooth. The better-developed posteroloph is tall and has both labial and lingual extensions. The anterolingual reentrant is shallower than the posterolingual one. This tooth has a single root.

M1–The occlusal outline of these teeth is subquadrate (Fig. 3E–M). The subequal protocone and hypocone are connected by a short and slightly oblique endoloph. The anteroloph and the protoloph are fused at a very early stage of wear and the paraflexus is absent in all specimens. The metaflexus is usually distinct, but it obliterates late in wear as the metaloph and the posteroloph fuse (Zahleh 26). These teeth have three roots (two labial and a lingual).

M2–The posteroloph of these teeth is more posteriorly directed than on the M1 (Figs 3N–R and 4A-F). The paraflexus is obliterated by wear about as rapidly as in the M1 and only the less worn specimen (Zahleh 24, Fig. 4A–C) shows a slight indentation along its anterolabial margin. Two specimens (Zahleh 27, Zahleh 22, Fig. 4G–L) have been tentatively identified as M2. These teeth are three-rooted.

M3–The posterior side of the tooth is reduced and, therefore, the hypocone is somewhat smaller than the protocone (Fig. 4M–O). This tooth lacks the paraflexus and its metaflexus is reduced to a constriction on the posterolabial side of the tooth. The hypoflexus is anterolabially directed and the hypostriid is deep.

#### Comparisons

The main morphological differences between *Proafricanomys* and *Africanomys* spp. are found in the dp4, which in the former taxon shows the anteroconid, the metaconid more posteriorly located and the metalophulid II anterolingually directed instead of anteriorly oriented. Another important difference between the two taxa concerns the development of the metaflexus on the upper molars and permanent premolar. Thus, whereas both the paraflexus and metaflexus are obliterated very early in wear in *Africanomys*, *Proafricanomys* conserves a distinct metaflexus until an advanced degree of wear. Detailed comparisons with all taxa belonging to the genus *Africanomys* are available as online supplementary content (Supplementary Information 2).

#### Cladistic analysis

The cladistic analysis including all valid species of Ctenodactylinae (as determined in López-Antoñanzas and Knoll^1^ and subsequent works^16^,^17^) with less than 50% of missing data, has produced a single most parsimonious tree with a length of 85 and a relatively low degree of homoplasy (CI = 0.635; RI = 0.856).

**Figure 5:**
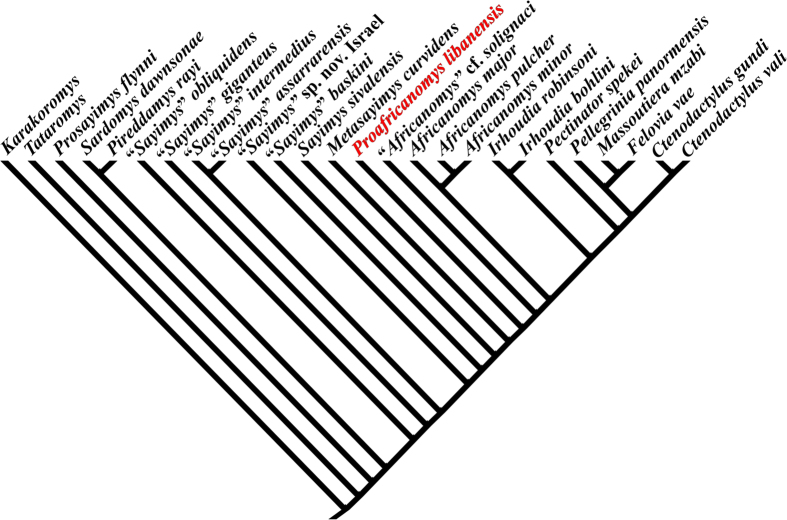
Single most parsimonious tree generated by the cladistic analysis of all the valid species of Ctenodactylinae with less than 50% of missing data (matrix in Supplementary text S3).

Our results (Fig. 5) show that *Proafricanomys libanensis* is more derived than *Metasayimys curvidens* but more basal than *Africanomys* cf. *solignaci*. According to our topology, the latter species does not belong to the genus *Africanomys*. This result is in agreement with the primitive morphology displayed by the cheek teeth of *Africanomys* cf. *solignaci* when compared with *Africanomys* spp. In fact, the morphology of *Africanomys* cf. *solignaci* recalls that of Proafricanomys. Indeed, the dp4 of *Africanomys* cf. *solignaci* has an anteroconid, the metaconid posteriorly located and the metalophulid II anterolingually directed instead of anteriorly oriented, and shows the metaflexus on the upper molars. It is likely that these two species will group as sister taxa when additional material will allow a more comprehensive scoring. Thus, *Africanomys* cf. *solignaci* is a potential second species of the genus *Proafricanomys*.

Our results also reveal that *Metasayimys curvidens* is intermediate in phylogenetic position between a *Sayimys*-grade group of ctenodactylines and *Proafricanomys libanensis*. *Metasayimys* is only one node up-tree with respect to *Sayimys sivalensis*. The morphology of *Metasayimys* soundly recalls that of *Sayimys sivalensis*, whereas it is quite different from that of *Africanomys* spp. For instance, *Metasayimys curvidens* has metalophulid I on the dp4: it is lost in the more derived *Proafricanomys* and *Africanomys* spp. In addition, *Metasayimys curvidens* has the posterior side of the M3 unreduced, whereas it is reduced in *Proafricanomys* and *Africanomys.*

## Discussion

**Figure 6:**
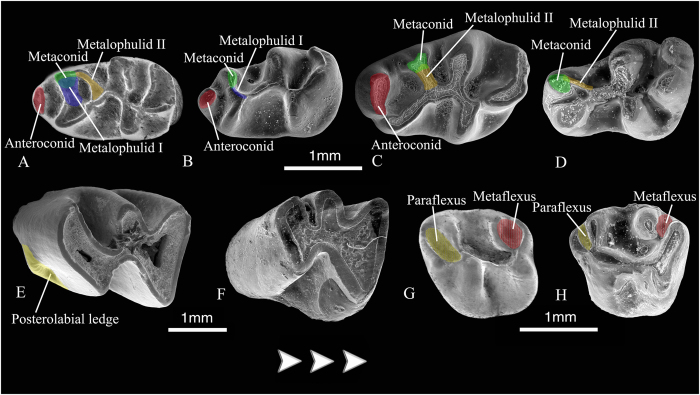
Evolutionary trends in the pattern of the cheek teeth in Ctenodactylinae. **(A–D)** evolution of the dp4. **(A)** dp4 of *Sayimys assarrarensis* with the plesiomorphies of having an anteroconid and both metalophulid II and metalophulid I; **(B)** dp4 of *Metasayimys sivalensis* with the plesiomorphy of having an anteroconid and the synapomorphy of having metalophulid I but not metalophulid II; **(C)** dp4 of *Proafricanomys libanensis* nov. gen. nov. sp. with the plesiomorphy of having an anteroconid and the synapomorphy of having metalophulid II but not metalophulid I; **(D)** dp4 of *Africanomys pulcher* with the synapomorphies of lacking an anteroconid and metalophulid I. **(E–F)** lower molars. **(E)** m3 of *Proafricanomys libanensis* with the plesiomorphy of having a well-developed posterolabial ledge. **(F)** m3 of *Africanomys pulcher* with the synapomorphy of lacking the posterolabial ledge. **(G–H)** DP4. **(G)** DP4 of *Sayimys assarrarensis* with the plesiomorphies of having a well-developed paraflexus and metaflexus; **(H)** DP4 of *Africanomys pulcher* with the synapomorphies of having small paraflexus and metaflexus. Images obtained from Scanning Electron Microscopy (SEM).

The evolution of the dp4 in the Ctenodactylinae is characterized by the loss of the anteroconid and the metalophulid I, the connection of the metaconid to the metalophulid II, the shift anteriorly of the metaconid and the protoconid, and the subsequent lengthening of the posterior arm of the protoconid (Fig. 6A–D). The metalophulid I is lost prior to the disappearance of the anteroconid. The presence of the metalophulid I (6(0 → 1)) is a basal synapomorphy of Ctenodactylinae that is reversed (6 (1 → 0)) in the common ancestor of *Proafricanomys* and the more derived members of the group (Fig. 6A–D). The presence of the anteroconid on the dp4 (7(0 → 1)) is a basal synapomorphy that is lost at the level of *Africanomys*. *Africanomys* and the more derived Ctenodactylinae share the synapomorphy of having this structure reversed (7(1 → 0)) (Fig. 6D). The loss of the anteroconid on the dp4 is a consequence of the shift of the metaconid anteriorly (8(1 → 2)). Thus, *Proafricanomys*, which has already lost the metalophulid I, still maintain the anteroconid because even though the protoconid has already moved anteriorly, the metaconid is still lingually situated (8(1)), near the entoconid (Fig. 6C). The metaconid is connected to the metalophulid I (9(0 → 1)) in the most primitive ctenodactylines (Fig. 6A–B). *Proafricanomys* and the more derived ctenodactylines share the synapomorphy of having the metaconid joined to the metalophulid II (9(1 → 0)) (Fig. 6C–D). Thus, the most derived Ctenodactylinae show to some extent a return to deciduous premolar morphology that is typical for primitive ctenodactylids.

With regard to the permanent premolars, the clade *Africanomys* spp. shares the synapomorphy of having a vestigial hypoconid (11(1 → 0)), which is again a reversal. Unfortunately, the characters of the p4 are missing in *Proafricanomys*, *Irhoudia*, *Pellegrinia*, and all extant Ctenodactylinae but *Pectinator spekei*.

*Proafricanomys* and *Africanomys* are characterized by a wide open V-shaped mesoflexid that is clearly shorter than the metaflexid on the lower molars. The large sample of worn and unworn cheek teeth of *Africanomys pulcher* that we have examined allows correlation of this morphology with the presence of a metalophulid II that fuses with the metalophulid I very early through wear (15(0 → 1)). However, this character appears in the evolution of Ctenodactylinae long before the emergence of the first *Proafricanomys* and *Africanomys* and it is lost three times (in *Sayimys intermedius*, *Sayimys sivalensis*, and *Irhoudia* spp.+more derived Ctenodactylinae).

The weakening of the posterolabial ledge (18(1 → 0)) on the lower molars is an evolutionary reversal shared by all Ctenodactylinae more derived than *Proafricanomys* (Fig. 6F). *Proafricanomys* is the most derived taxon that has a well-developed posterolabial ledge on the lower molars (Fig. 6E).

On the DP4 and upper molars, reduction of the metaflexus and paraflexus very early in wear, and their subsequent loss, is another feature that characterizes the most derived members of the ctenodactylines (Fig. 6G–H). With regard to the DP4, *Proafricanomys* and *Africanomys* cf. *solignaci* are the most derived ctenodactylines that still have a well-developed metaflexus. *Africanomys* spp. and *Irhoudia* spp. have the metacone connected very labially to the posteroloph (or isolated from it) so that the metaflexus is much reduced (24(0 → 1)) and the most derived ctenodactylines lose it early in wear (24(1 → 2)).

The reduction and subsequent loss of the paraflexus and the metaflexus is evidenced earlier on the upper molars than on the DP4. In addition, the loss of the paraflexus preceeds that of the metaflexus. More specifically, the most primitive taxon with obliterated paraflexus (29(0 → 1)) is *Sayimys baskini*, whereas the most primitive species with obliterated metaflexus (31(0 → 1)) is *Metasayimys curvidens*.

An evolutionary trend toward a simplification of the morphology of the P4 is also characteristic of the Ctenodactylinae. However, this simplification starts prior to the appearance of *Proafricanomys* (*Sayimys sivalensis *+ more derived ctenodactylines (26(0 → 1); 28(0 → 1)) and with *Sayimys baskini* + more derived ctenodactylines (27(0 → 1))).

Finally, the reduction of the posterior side of the M3 (34(0 → 1)) is another synapomorphy shared by *Sayimys* nov. sp. from Israel^2^, *Sayimys baskini*, *Sayimys sivalensis*, *Proafricanomys*, *Africanomys* cf. *solignaci*, and *Africanomys* spp. This synapomorphy is then lost in *Irhoudia* spp.+more derived ctenodactylines.

The discovery and phylogenetic analysis of *Proafricanomys* better defines the evolutionary history of *Africanomys*. *Proafricanomys* nov. gen. nov. sp. is the 'missing link' that sheds light on the transition from the paraphyletic '*Sayimys*' to the clade *Africanomys*. This pivotal taxon shares a mosaic of characters with both the most primitive and more derived members of the subfamily. Thus, as in the most plesiomorphic ctenodactylines, the dp4 of *Proafricanomys* has a distinct anteroconid and the metaconid lingually located, near the entoconid and its lower molars show a well-developed posterolabial ledge. In addition, *Proafricanomys* shares with the most derived members of the subfamily the synapomorphic loss of the metalophulid I on the dp4 and the obliteration of the metaflexus early in wear on the upper molars.

As detailed above, the evolutionary stage of *Proafricanomys* is comparable to that of *Africanomys* cf. *solignaci* from Sheikh Abdallah (Egypt). As this latter taxon is Late Miocene (MN 9, ~11 Ma) in age^16^ it is plausible that the layer horizon from which *Proafricanomys libanensis* comes is several million years older than previously thought, namely Tortonian rather than Messinian. This is corroborated by the co-occurrence of *Progonomys* sp., which is usually correlated with MN 10 and/or MN 11^18^. However, a detailed study of the entire micromammal fauna of the site is necessary before assessing its age with confidence.

## Conclusion

The new species of ctenodactyline from the Late Miocene of Lebanon, *Proafricanomys libanensis*, is situated at a pivotal phylogenetic position between a paraphyletic group of *Sayimys*-like species and the more derived *Africanomys*. As such, it provides data unavailable so far regarding the expected dental morphology of the ancestor of the African ctenodactylines, including the crown group, but with the exception of *Metasayimys curvidens*. Although not from Africa or even the African plate, it is the sister-species of the clade composed of all but one of the ctenodactylines that have originated in Africa (no Asian species are present in this clade and *Pellegrinia* represents only a brief extra-African off-shoot). In other words, *Proafricanomys libanensis* may be seen as the most evolved of the non-African ctenodactylines. The phylogenetical position of *Proafricanomys libanensis* argues in favour of an independent origin for *Africanomys* and *Metasayimys* and sustains the hypothesis that this subfamily dispersed more than once from Asia into Africa.

The evolutionary stage and relationships of *Proafricanomys libanensis* suggest that the deposition of the 'Zahleh Formation' might have been initiated as early as the Tortonian, not the Messinian as currently believed. The discovery of *Proafricanomys libanensis* demonstrates how key to our understanding of mammalian phylogeographic history is continued field work in the large but often neglected Arabian Plate.

## Methods

### Specimens

We examined the following: skulls of extant *Massoutiera mzabi* (71064, 71149, 71154, 71156, 71157, 71152, 71155, 71150, 71158, 71161, 71163, 71164, 71151, 71162, 71159, 37735, 37736, 37737, 37738, 37739 in the MB and C.G.1960-3741, C.G.1959-93, C.G.1960-3812, C.G.1959-92, C.G.1989-29, C.G.1912-322, C.G.1953-381, C.G.1955-3, C.G.2000-686 in the MNHN), *Felovia vae* (41239, 41242, 4124 in the MB and CG-1994-612, CG-1994-613, C.G.1995-3157 in the MNHN), *Ctenodactylus gundi* (15515, 15516, 71186, 25640, 37765, 1302, 71177, 71188, 71185, 20721, 2784, 71179, 71181, 71187, 71182 in the MB and C.G.1963-921, C.G.1975-303, C.G.1975-304, C.G.1975-305, C.G.1975-306, C.G.1975-307, C.G.1975-309, C.G.1975-308, C.G.1975-306, C.G.1975-304, C.G.1905-437, C.G.1991-1298, C.G.1991-1316, C.G.1993-1680, C.G.2007-329 in the MNHN), *Ctenodactylus vali* (C.G.1952-664, C.G.1952-666, C.G.1952-668, C.G.1951-389, C.G.1953-787 in the MNHN), *Pectinator spekei* (26563, 71171, 71169, 71153, 37977, A2636, 3935, 71165, 71166, 71167, 71175, 71170, 71172, 71174, 71173 in the MB and C.G.1895-461, C.G.1895-459, C.G.1895-460, C.G.1986-240, C.G.1978-263, C.G.1978-264, C.G.1978-265, C.G.1978-266, C.G.1978-267, C.G.1981-504, C.G.1960-3744, C.G.1960-3783 in the MNHN); isolated teeth, maxillary fragments, and mandible fragments of the following extinct species: *Prosayimys flynni* (casts of Z295, Z307, Z317, Z312, Z308, Z309, Z311, Z313, Z310, Z316, Z294, Z292, Z287, Z289, Z293, Z290, Z306, Z296, Z291, Z305, Z288, Z303, Z304, Z297 in RLA’s personal collection), *Sayimys assarrarensis* (AS21-1023, AS21 1001, AS21 1002, AS8 1001, AS21 1008, AS8 1000, AS21 1005, AS21 1004, AS21 1018, AS21 1017, AS8 1003, AS21 1016, AS21 1024, AS21 1026, AS21 1028, AS8 1002, AS21 1025 in the MNHN), *Sayimys giganteus* (KSK1-100 to KSK1-102; KSK2-100 to KSK2-104; HJ-100 to HJ-108 in the MNHN), *Sayimys intermedius* (TMA 100, TMA 101 in the MNHN) and from Chios Island, Greece (THA91-01 to THA91-03, THA91-10 to THA91-14, THA91-21 to THA91-25, THA91-28 to THA91-31; THA93-26, THA93-27, THA93-04 to THA93-09; THA93-15 to THA93-20, THA93-32 to THA93-36 in the MNHN), *Sayimys chinjiensis* (= *Sayimys* *sivalensis*) (casts of Y-GSP 634/45186, Y-GSP 634/45183, Y-GSP 634/45187 in RLA’s personal collection), *Metasayimys curvidens* (Ben Mel 1357, Ben Mel 1353, Ben Mel 1354, Ben Mel 1371, CBR-188, CBR-189, CBR-191, CBR-192, CBR-194, CBR-197, CBR-198, CBR-199, CBR-205, CBR-207, CBR-208, CBR-209, CBR-210, CBR-211, CBR-212, CBR-214, CBR-215, CBR-218-CBR-222, CBR-225-CBR-230, CBR-234-CBR-236, CBR-238, CBR-248, CBR-249, CBR-251, CBR-252, CBR-257, CBR-261, CBR-268, CBR-271, CBR-274, CBR-281-CBR-283, CBR-287, CBR-289, CBR-291, CBR-294, CBR-296, CBR-300- CBR-302, CBR-304-CBR-309, CBR-311, CBR-315-CBR-328, CBR-331, CBR-335, CBR-337, CBR-338, CBR-347, CBR-350, CBR-352, CBR-356-CBR-359, CBR-360, CBR-363, CBR-370, CBR-373-CBR-384, CBR-387-CBR-389, CBR-396, CBR-398, CBR-399, CBR-460-CBR-462, CBR-465, CBR-469, CBR-471-CBR-475, CBR-477-CBR-479, CBR-481, CBR-484, CBR-487, CBR-488, CBR-531, CBR-534, CBR-535, CBR-537, CBR-551, CBR-553-CBR-556, CBR-558, CBR-559, CBR-562, CBR-565, CBR-566, CBR-568, CBR-571-CBR-574 CBR-577, CBR-578, CBR-580, CBR-696, CBR-697 in the MNHN), *Africanomys pulcher* (Ben Mel 1356; Ben Mel 1367, Ben Mel 1369, CBR-2-CBR-5, CBR-7-CBR-10, CBR-12, CBR-13, CBR-16, CBR-19, CBR-23, CBR-27, CBR-28, CBR-30, CBR-49-CBR-57, CBR-59, CBR-61, CBR-62-CBR-65, CBR-66, CBR-68, CBR-69-CBR-72-CBR-74, CBR-76-CBR-78, CBR-84-CBR-87, CBR-89, CBR-90, CBR-91, CBR-96-CBR-98, CBR-101, CBR-106, CBR-107, CBR-113, CBR-116, CBR-117, CBR-127-CBR-135, CBR-144, CBR-145, CBR-147-CBR-151, CBR-153-CBR-164, CBR-166, CBR-168, CBR-169, CBR-170, CBR-172, CBR-174, CBR-180, CBR-181, unnumbered CBR in the MNHN).

The new specimens have been described and compared with all the known species of Ctenodactylinae as established by López-Antoñanzas and Knoll^1^ and a few erected subsequently^16^,^17^. However, a detailed comparison is only carried out with the species considered as belonging to the genus *Africanomys*, the closest relatives to the new Lebanese taxon. First, second, and third lower molars are designated as m1, m2, and m3 respectively and first, second, and third upper molars as M1, M2, and M3. Lower and upper permanent premolars are designated as p4 and P4, respectively, and lower and upper deciduous premolars as dp4 and DP4. The terminology used in the tooth descriptions follows the rodent dental terminology of Baskin^19^ and López-Antoñanzas and Knoll^1^. The occlusal measurements (greatest length and greatest width; Supplementary Table S1) of the teeth of the new taxon from Lebanon have been obtained with a Nikon digital counter CM-6S measuring device.The stratigraphical range of all the species of Ctenodactylinae mentioned in this work is summarized in Supplementary Table S2.

### Cladistic analysis

The cladistic analysis carried out in this work treated as ingroup all the valid species of Ctenodactylinae known to date. *Karakoromys* and *Tataromys*, basal ctenodactylid genera according to the phylogenetic analysis of Wang^20^ and Vianey-Liaud *et al.*^17^ are selected as outgroup. Recent works^21^ have shown that numerous missing entries in a few taxa are most detrimental to a phylogenetic analysis as the information needed to place these taxa in the tree is depleted and, therefore, nodes are meant to collapse in the consensus tree. Accordingly, we have excluded from our analysis five taxa (*Helanshania deserta*, *Sardomys antoniettae, Akzharomys mallos*, *Africanomys kettarati*, and *A. bahariyaensis* with over 50% of missing data. A total of 34 phylogenetically informative dental characters have been coded (Supplementary Text S2). Of these, 17 characters are binary, whereas 17 are multistate. All the latter characters have two or three derived states. Owing to the lack of a priori information, all characters were unordered and equally weighted (Fitch optimality criterion). The data matrix (Supplementary Text S3) was built under Mesquite 2.6^22^ and processed with TNT^23^ with the 'traditional search' option (using TBR).

### Nomenclatural acts

This published work and the nomenclatural acts it contains have been registered in ZooBank, the proposed online registration system for the International Code of Zoological Nomenclature (ICZN). The ZooBank LSIDs (Life Science Identifiers) can be resolved and the associated information viewed through any standard web browser by appending the LSID to the prefix ‘ http://zoobank.org/’. The LSIDs for this publication are urn:lsid:zoobank.org:pub:2EE2A1F6-0F75-4A10-8C47-90C1463FB72F, urn:lsid:zoobank.org:act:2884F6CA-B818-4EC6-BA74-87EE9C30D17A and urn:lsid:zoobank.org:act:ADF4D62D-BBF5-443B-B29B-153FB229E4E7.

## Additional Information

**How to cite this article**: López-Antoñanzas, R. *et al.* First Miocene rodent from Lebanon provides the 'missing link' between Asian and African gundis (Rodentia: Ctenodactylidae). *Sci. Rep.* **5**, 12871; doi: 10.1038/srep12871 (2015).

## Supplementary Material

Supplementary Information

## Figures and Tables

**Figure 1:**
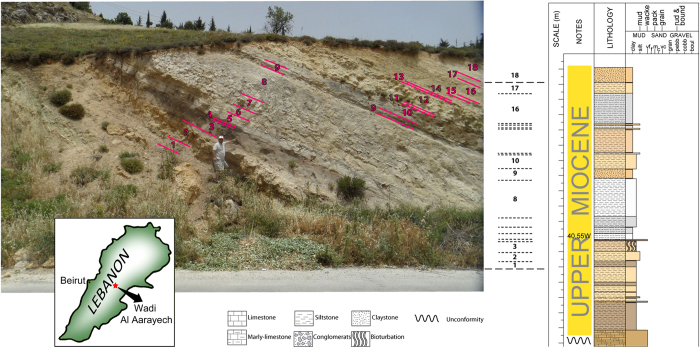
Exposure in the Zahleh area (Lebanon) with stratigraphic section (right) and geographic location (inset). *Proafricanomys libanensis* nov. gen. nov. sp. was recovered from layer (nº3). The map and the section were generated by us using the software Adobe illustrator (Adobe Systems Inc.). Photograph by FK.

## References

[b1] López-Antoñanzas R. & Knoll F. A comprehensive phylogeny of the gundis (Ctenodactylinae, Ctenodactylidae). J. Syst. Palaeontol. 9, 379–398 (2011).

[b2] López-Antoñanzas R., Gutkin V., Rabinovich R., Calvo R. & Grossman A. The rodent fauna from the Early Miocene of the Rotem Basin (Israel): African, Asian, both or neither? J. Vertebr. Paleontol., Program and Abstracts. 170 (2014).

[b3] López-Antoñanzas R. & Sen S. Ctenodactylids from the Lower and Middle Miocene of Saudi Arabia. Palaeontology 47, 1477–1494 (2004).

[b4] López-Antoñanzas R. *Neogene Ctenodactylidae, Thryonomyidae, and Zapodidae (Rodentia) from the Middle East: systematics, phylogeny, biostratigraphy, palaeogeography, and palaeoecology*. PhD dissertation, Muséum national d’Histoire naturelle, Paris (2004).

[b5] Sen S. & Thomas H. Découverte de rongeurs dans le Miocène moyen de la Formation Hofuf (Province du Hasa, Arabie Saoudite). C. R. Somm. Soc. Géol. France 21, 34–37 (1979).

[b6] De Bruijn H. & Whybrow P. J. A Late Miocene rodent fauna from the Baynunah Formation, Emirate of Abu Dhabi, United Arab Emirates. Proc. Kon. Nederl. Akad. Wetensch. B 97, 407–422 (1994).

[b7] De Bruijn H. Superfamily Ctenodactyloidea. The Miocene land Mammals of Europe (eds Rössner G. H. & Heissig K.) 263–266 (Dr. Friedrich Pfeil, München, 1999).

[b8] Kraatz B., Bibi F. & Hill A. New rodents from the Late Miocene of the United Arab Emirates. J. Vertebr. Paleontol. 29, 129A (2009).

[b9] Kraatz B. P., Bibi F., Hill A. & Beech M. A new fossil thryonomyid from the Late Miocene of the United Arab Emirates and the origin of African cane rats. Naturwissenschaften 100, 437–449 (2013). 23625517 10.1007/s00114-013-1043-4

[b10] Kansou M. Découverte de vertébrés pontiens au Liban dans la plaine de la Békaa. Bull. Sci. Cons. Acad. RPF Yougoslavie 6, 65 (1961).

[b11] Malez M. & Forsten A. *Hipparion* from the Bekaa valley of Lebanon. Geobios 22, 665–670 (1989).

[b12] Dubertret L. & Vautrin H. Sur l’existence du Pontien lacustre en Syrie et sur sa signification tectonique. C. R. Heb. Acad. Sci., Paris 206, 69–71 (1938).

[b13] Haj-Chahine T. *Etude sédimentologique des formations lacustres néogènes de la région de Zahleh, Liban*. PhD dissertation, Université Paris VI, Paris (1973).

[b14] Malez M. & Crnolatac I. Relations stratigraphiques et paléontologiques du gisement de la faune à Hipparion près de Kefraya (Békaa, Liban). Bull. Sci. Cons. Acad. RPF Yougoslavie 6, 97–98 (1961).

[b15] Walley C. D. The lithostratigraphy of Lebanon: a review. Lebanese Sci. Bul. 10, 81–108 (1997).

[b16] Mein P. & Pickford M. Vallesian rodents from Sheikh Abdallah, Western Desert, Egypt. Historical Biol. 22, 224–259 (2010).

[b17] Vianey-Liaud M., Gomes-Rodrigues H. & Marivaux L. A new Oligocene Ctenodactylinae (Rodentia: Mammalia) from Ulantatal (nei Mongol): new insight on the phylogenetic origins of the modern Ctenodactylidae. Zool. J. Linn. Soc. 160, 531–550 (2010).

[b18] Mein P., Martín Suárez E. & Agustí J. *Progonomys* Schaub, 1938 and *Huerzelerimys* gen. nov. (Rodentia); their evolution in Western Europe. Scripta Geol. 103, 41–64 (1993).

[b19] Baskin J. A. Systematic revision of Ctenodactylidae (Mammalia, Rodentia) from the Miocene of Pakistan. Palaeovertebrata 25, 1–49 (1996).

[b20] Wang B. Y. The mid-Tertiary Ctenodactylidae (Rodentia, Mammalia) of Eastern and Central Asia. Bull. Am. Mus. Nat. Hist. 234, 1–88 (1997).

[b21] Prevosti F. J. & Chemisquy M. A. The impact of missing data on real morphological phylogenies: influence of the number and distribution of missing entries. Cladistics 26, 326–339 (2010). 10.1111/j.1096-0031.2009.00289.x 34875786

[b22] Maddison W. P. & Maddison D. R. Mesquite: a modular system for evolutionary analysis, Version 2.6. Mesquite Project, Vancouver (2009).

[b23] Goloboff P., Farris J. & Nixon K. C. TNT, a free program for phylogenetic analysis. Cladistics 24, 774–786 (2008).

