# Supplementary material for: First Miocene rodent from Lebanon provides the 'missing link' between Asian and African gundis (Rodentia: Ctenodactylidae)
Source: Sci Rep. 2015 Aug 7;5:12871. doi: 10.1038/srep12871 (PMC4528195; doi:10.1038/srep12871)
Supplement: Supplementary Information [file srep12871-s1.pdf]

SUPPORTING INFORMATION

First Miocene rodent from Lebanon provides the 'missing link' between Asian and African gundis (Rodentia: Ctenodactylidae)

Raquel López-Antoñanzas[1,2], Fabien Knoll[1,2,3], Sibelle Maksoud[4], Dany Azar[5]

[1]School of Earth Sciences, University of Bristol, Bristol, United Kingdom

[2]Departamento de Paleobiología, Museo Nacional de Ciencias Naturales-CSIC, Madrid, Spain

[3]School of Earth, Atmospheric and Environmental Sciences, University of Manchester, Manchester, United Kingdom

[4] UMR 6538 Domaine Océanique, Université de Bretagne Occidentale, Brest, France

[5]Département des Sciences naturelles, Université Libanaise, Fanar, Lebanon

Supplementary Figure S1 | Surface rendering of the holotype (Zahle 13) of *Proafricanomys libanensis* nov. gen. nov sp.

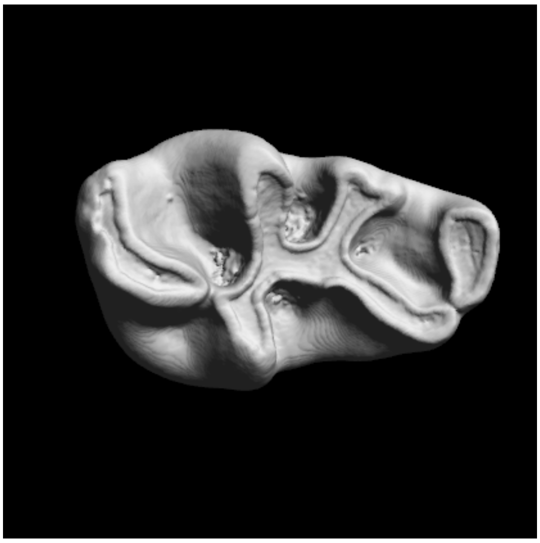

Supplementary Figure S2 | Surface rendering of the P4 (Zahle 6) of *Proafricanomys libanensis* nov. gen. nov sp.

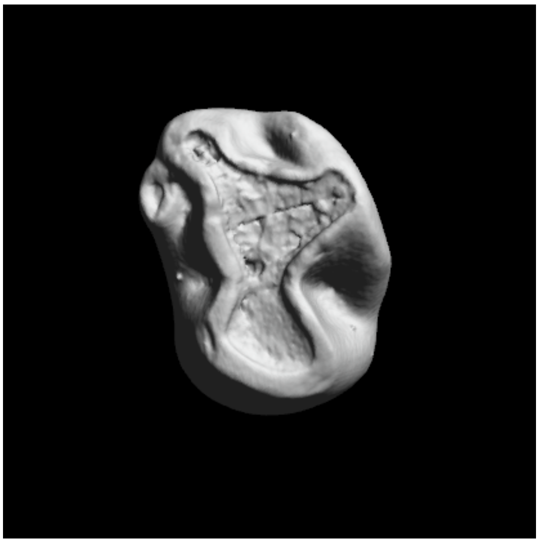

Supplementary Table S1 | Occlusal measurements (mm) of the teeth of *Proafricanomys libanensis* nov. gen. nov. sp. All measurements represent greatest length and greatest width.

	Specimen	Length	Width
dp4	Zahleh 13	2.454	1.578
m1-2	Zahleh 3 (broken)	-	1.905
m1-2	Zahleh 10 (broken)	-	-
m3	Zahleh 28	2.891	2.219
DP4	Zahleh 7 (broken)	2.027	1.927
P4	Zahleh 6	1.314	1.706
M1	Zahleh 26	2.196	2.679
M1	Zahleh 25	2.222	2.457
M1	Zahleh 47	2.372	2.342
M2	Zahleh 46	2.726	2.435
M2	Zahleh 23 (broken)	2.558	2.461
M2	Zahleh 12	3.344	2.550
M2	Zahleh 24	2.457	2.501
M2	Zahleh 9	2.869	2.753
M2-M3	Zahleh 27	2.523	2.572
M2-M3	Zahleh 22	2.780	2.647
M3	Zahleh 11	2.630	2.569

Supplementary Table S2 | Stratigraphical range of all fossil Ctenodactylinae mentioned in this work.

Taxon	Age	References
Prosayimys flynni Baskin, 1996	latest Oligocene	1,2
Sardomys dawsonae De Bruijn & Rümke, 1974	Early Miocene (MN1-MN2)	3
Pireddamys rayi De Bruijn & Rümke, 1974	Early Miocene (MN1-MN2)	3
Sayimys obliquidens Bohlin, 1946	Early Miocene (MN 3-MN4)	2,4,5
Sayimys giganteus López-Antoñanzas, Sen & Sarac, 2004	Early Miocene (MN3-MN4)	2,6
Sayimys baskini López-Antoñanzas & Sen, 2003	Early Miocene (MN3)	2,7,8,9
Sayimys intermedius (Sen &Thomas, 1979)	Early-Middle Miocene (MN4-MN6)	2,7, 8, 9, 10, 11,12,13
S. assarrarensis López-Antoñanzas & Sen, 2004	Early Miocene (MN5)	2,10
Sayimys sivalensis (Hinton, 1933)	Middle-Late Miocene (MN6-MN10)	2,14,15,16,17, 18,19,20, 21
Metasayimys curvidens Lavocat, 1961	Middle Miocene (MN6)	22, 23,24,25,26,27
Africanomys pulcher Lavocat, 1961	Middle Miocene (MN6-MN7)	22, 23,24,25,26,27
Africanomys major Jaeger, 1977	Middle-Late Miocene (MN6-MN9)	22,26,28,29,30,31
Africanomys minor Jaeger, 1977	Middle-Late Miocene (MN6-MN9)	22,26,31
Africanomys solignaci (Robinson & Black, 1973)	Late Miocene (MN9)	31
Proafricanomys libanensis nov. gen. nov. sp. López-Antoñanzas, Knoll, Maksoud & Azar, this work	Late Miocene (MN10-MN11?)	This work
Irhoudia robinsoni Jaeger, 1977	Late Miocene (MN12)	22,26
Irhoudia bohlini Jaeger, 1971	Late Pliocene-Early Pleistocene	22,27,32
Pellegrinia panormensis De Gregorio, 1887	probably Early Pleistocene	33,34,35,36,37,38,39

Supplementary Text S1| Comparisons

1. Comparison with *Africanomys pulcher* Lavocat, 1961

The holotype of this species (Ben. Mel.1375) is a right DP4 from the Middle Miocene of Beni-Mellal, which is housed in the MNHN. Jaeger[27] synonymized *Africanomys incertus* (*partim*) with *A. pulcher,* an action with which we concur. Additional material of this taxon has been recovered from the Middle Miocene site of Jebel Rhassoul, Morocco[24-25]. Furthermore, the presence of *Africanomys* cf. *pulcher* has been mentioned by Benammi[25] from the Middle Miocene localities of Azdal 1 and 3 (Aït Kandoula, Morocco).

The main morphological differences between *Proafricanomys* and *Africanomys pulcher* are found in the dp4, which in *Proafricanomys* has an anteroconid, the metaconid very much posteriorly located and the metalophulid II anterolingually directed instead of anteriorly-oriented. The dp4 of *A. pulcher* lacks the anteroconid and has both metalophulid II and metaconid on the anterior side of the tooth. Another important difference between the two taxa is the development of the metaflexus on the upper molars and permanent premolar. Thus, whereas both the paraflexus and metaflexus are obliterated very early in wear in *A. pulcher*, *Proafricanomys* conserves a distinct metaflexus until an advanced degree of wear.

2. Comparison with *Africanomys major* Jaeger, 1977

This species was defined on the basis of 14 isolated dp4, 28 mandible fragments and 18 maxillary fragments from the locality Pataniak 6 in Jebel Irhoud, Morocco[26]. This locality has been considered Middle Miocene in age[28]. The holotype (P6-141) is a mandible fragment with p4-m3 housed in the SGM[4]. Even though additional material of this taxon has been cited from the Upper Miocene of Sheikh Abdallah, Egypt[29-30], a recent thorough report on the micromammals of this area has failed to record *Africanomys major*[31]. The morphology of the dp4 of *Proafricanomys* and that of *A. major* are very different. *Proafricanomys* is characterized by having a distinct anteroconid, the metalophulid II anterolingually directed and the metaconid more posteriorly located. In

Africanomys major the metalophulid II is anteriorly oriented and joins with the metaconid on the anterior side of the tooth. With regard to the upper molars, both paraflexus and metaflexus obliterate early in wear in *Africanomys major,* whereas *Proafricanomys* is characterized by a metaflexus that remains distinct into advanced wear.

3. Comparison with *Africanomys minor* Jaeger, 1977

Africanomys minor comes from the Middle Miocene locality Pataniak 6 (Jebel Irhoud, Morocco)[26]. *Africanomys* cf. *minor* has been recovered from the Upper Miocene of Sheikh Abdallah, Egypt[29,31].

There are important dental differences between *Africanomys minor* and the new species from Lebanon. First, the dp4 of *Proafricanomys* has a large and very distinct anteroconid that is absent in *A. minor,* and the metalophulid II is anterolingually directed and the metaconid much more posteriorly located than in the African taxon. In the upper molars of *Proafricanomys* the paraflexus is shallow and soon disappears by wear, whereas the metaflexus persists later in wear. In contrast, both the paraflexus and metaflexus disappear quickly through wear in *A. minor*. The cheek teeth of *Proafricanomys* are larger than those of *A. minor*.

4. Comparison with *Africanomys kettarati* Jaeger, 1977

This species was defined on the basis of six isolated teeth from the Upper Miocene locality Oued Zra (Middle Atlas, Morocco). Its holotype (OZ-36) is an isolated M1-M2 housed in UM (B. Marandat pers. comm. 2014). Additional material of this species has been recovered from the Upper Miocene of Bou Hanifia 5, Algeria[40]. Unfortunately, no premolars of *Africanomys kettarati* have been found to date. On the lower molars, the posterolophid of *A. kettarati* is constricted before reaching the triangular wear surface of the hypoconid and the tooth lacks a posterolabial ledge whereas those of *Proafricanomys* have the posterolophid unconstricted and the posterolabial ledge

well developed. The upper molars of *A. kettarati* have the metaflexus much less developed than those of *Proafricanomys*. The cheek teeth of *Proafricanomys* are larger than those of *A. kettarati*.

5. Comparison with *Africanomys bahariyaensis* Mein and Pickford, 2010

The holotype of this taxon (SA 32) is a mandible with the incisor and p4-m3. It comes from the Late Miocene locality 172 of Sheikh Abdallah (Egypt)[31]. Some upper teeth from Bou Hanifia 5 (Algeria), which are consistent in size with the holotype of *A. bahariyaensis,* have been tentatively assigned to this taxon[31]. As the upper molars of *A. bahariyaensis* are not known definitively, comparisons are not made with *Proafricanomys*. Comparison of the lower deciduous and permanent premolars of these two taxa is not possible either, due to the lack of dp4 of *A. bahariyaensis* and the absence of p4 belonging to *Proafricanomys.* However, the m3 of *Proafricanomys* has the protoconid more developed than the hypoconid. Furthermore, the anterior side of the tooth is quite wider than the posterior one, whereas this is not observed on the lower molars of *A. bahariyaensis*. The cheek teeth of *A. bahariyaensis* are also larger than those of *Proafricanomys* from Lebanon.

6. Comparison with *Africanomys* cf. *solignaci* (Robinson and Black 1973)

The taxon *Testouromys solignaci* was named on the basis of a single third lower molar (holotype: T-3724), a single eroded first upper molar (T-3724) and a broken first lower molar (T-3802) from the Middle Miocene of Testour Beja (Tunisia)[41]. This material was considered insufficient to define a new genus and species[2,12] and was assigned to *Africanomys*[2,31]. Recently, 14 isolated cheek teeth, two incisors, and a maxillary fragment with DP4 from Sheikh Abdallah (Egypt) have been tentatively assigned to *Africanomys* cf. *solignaci*[31]. Regardless of the problematical taxonomic allocation, it has been included in our analysis because of its completeness and utmost interest.

Repository Abbreviations: CBR, Collection of Dr J. Braillon; C.G., Catalogue général; Y-GSP, Yale-Geological Survey of Pakistan, Quetta, Pakistan; MB, Museum für Naturkunde der Humboldt-Universität, Berlin, Germany; MNHN, Muséum national d'Histoire naturelle, Paris, France; SGM, Geological service of Rabat, Morocco; UM, Université des Sciences et Techniques du Languedoc, Montpellier, France; Z, Zinda Pir area, Pakistan.

Supplementary Text S2| Characters used in the phylogenetic analysis. Seventeen characters are binary whereas seventeen are multistate. The polarity of characters was determined by outgroup (*Karakoromys decessus* and *Tataromys plicidens*) comparison.

1. LM1: (0) 1-2.5mm; (1) 2.5-3.5mm; (2) >3.5mm.
2. (0) Rooted cheek teeth; (1) ever-growing cheek teeth.
3. (0) Brachyodont; (1) semi-hypsodont; (2) hypsodont.
4. p3: (0) present; (1) absent.

dp4

5. (0) With three main lophs; (1) bilobed.
6. (0) With metalophulid II; (1) with metalophulid II and metalophulid I; (2) with metalophulid II+Metalophulid I; (3) only with metalophulid I.
7. Anteroconid: (0) absent; (1) present.
8. Metaconid: (0) anterolingual; (1) lingual; (2) anterior.
9. Metaconid: (0) connected to the metalophulid II (1) connected to the metalophulid I
10. Posterolophid: (0) long; (1) shortened; (2) fused with the entoconid/hypolophid forming a single lobe.

p4

11. (0) with entoconid and hypoconid reduced to a cingulid; (1) with only a posterior cuspid (entoconid); (2) minute.
12. Shape in occlusal view (0) U-shaped pattern; (1) Y-shaped pattern.
13. Posterolabial ledge: (0) absent;(1) present.

m1-m2

14. Unworn teeth with: (0) four lobes; (1) three lobes; (2) two lobes.
15. Metalophulid II: (0) present; (1) in early wear fused with metalophulid I; (2) absent.
16. Mesoflexid: (0) shorter than the metaflexid; (1) equal or longer than the metaflexid.
17. Metaflexid: long (0); (1) short; (2) absent.
18. Postero-labial ledge: (0) absent; (1) present.
19. Hypertrophy of the protoconid: (0) no; (1) yes.
20. Cement (on the m2-m3): (0) absent; (1) present.
21. (0) m2 longer than m3; (1) m2 equal in length with m3; (2) m2 shorter than m3.
22. Posterolophid: (0) well-developed; (1) absent.

I1

23. Longitudinal groove: (0) present; (1) weak; (2) absent.

DP4

24. Metaflexus: (0) long; (1) short; (2) absent at early stages of wear.
25. Paraflexus: (0) long; (1) short; (2) absent at early stages of wear.

P4

26. (0) as wide or wider than M1; (1) reduced but with small synclines; (2) with vestigial or without synclines; (3) minute; (4) absent.

27. (0) with vestigial anteroloph, protoloph, metaloph, and posteroloph but with protoloph and metaloph still distinct; (1) anteroloph short and one connection between metacone and protocone; (2) anteroloph absent, and one connection between metacone and protocone.
28. (0) with vestigial anteroloph, protoloph, metaloph, and posteroloph but with protoloph and metaloph still distinct; (1) posteroloph short and one connection between metacone and protocone; (2) anteroloph and posteroloph absent, and one connection between metacone and protocone.

M1-M2

29. Paraflexus: (0) well developed; (1) fused in early wear (2) absent.
30. Hypertrophy of the protocone: (0) no; (1) yes.
31. Metaflexus: (0) long; (1) short; (2) fused in early wear; (3) absent.
32. Hypoflexus: (0) deep; (1) shallow; (2) absent.
33. Cement: (0) absent; (1) present.

M3

34. Posterior lobe: (0) not reduced; (1) reduced.

Supplementary Text S3 | Character/taxon matrix. Character codings: 0, 1, 2 and 3, conditions of character; -, uncodable character; ?, character state uncertain.

	**1**	**2**	**3**	**4**	**5**	**6**	**7**	**8**	**9**	**10**	**11**	**12**	**13**	**14**	**15**	**16**	**17**	**18**	**19**	**20**	**21**	**22**	**23**	**24**	**25**	**26**	**27**	**28**	**29**	**30**	**31**	**32**	**33**	**34**
Karakoromys	0	0	0	0	0	0	0	0	0	0	0	0	0	0	0	0	0	0	0	0	0&1&2	0	0	0	0	0	0	0	0	0	0	1	0	1
Tataromys	2	0	0	0	0	0	0	0	0	0	0	0	0	0	0	0	0	0	0	0	0	0	0	0	0	0	0	0	0	0	0	1	0	0
Prosayimys flynni	0	0	0	?	0	1	1	1	1	0	1	0	0	0	0	0	0	1	0	0	1	0	?	0	0	1	1	1	0	0	0	0	0	0
Sardomys dawsonae	2	0	1	?	0	1	1	1	1	0	1	1	1	0	0	0	0	1	0	0	2	0	0	?	?	?	?	?	0	0	0	0	0	0
Pireddamys rayi	2	0	1	?	?	?	?	1	?	?	?	?	?	0	0	0	0	1	0	0	2	0	0	?	?	2	2	2	0	0	0	0	0	?
Sayimys obliquidens	0	0	1	?	0	1	1	1	1	0	1	1	1	0	0	0	0	1	0	0	1	0	0	0	0	1	1	1	0	0	0	0	0	0
Sayimys giganteus	1	0	1	0	0	2	1	1	1	0	1	1	1	1	1	0	0	1	0	0	1	0	?	0	0	1	1	1	0	0	0	0	0	0
Sayimys baskini	0	0	1	?	0	2	1	1	1	0	1	1	1	1	1	0	0	1	0	0	2	0	?	0	0	1	2	1	1	0	1	0	0	1
Sayimys intermedius	0	0	1	0	0	3	1	1	1	0	1	1	1	1	2	1	0	0&1	0	0	1	0	2	0	0	1	1	1	0	0	0	0	0	0
Sayimys assarrarensis	0	0	1	?	0	2	1	1	1	0	1	1	1	1	1	0	0	1	0	0	?	0	?	0	0	1	1	1	0	0	0	0	0	?
Sayimys sivalensis	0	0	1	?	0	3	1	1	1	0	1	1	1	1	2	1	0	0&1	0	0	2	0	?	0	0	1	2	2	1	0	1	0	0	1
Sayimys sp. nov. *Israel*	0	0	1	?	?	?	?	?	?	?	?	?	?	1	1	0	0	1	0	0	?	0	?	?	?	?	?	?	0	0	1	0	0	1
Metasayimys curvidens	0	0	1	0	0	2&3	1	1	1	0	1	1	0	1	1	0	0	1	0	1	2	0	0	0	0	3	2	2	1	0	2	0	1	0
Africanomys pulcher	0	0	1	0	0	0	0	2	0	0	0	1	0	1	1	0	0	0	0	0	1	0	?	1	1	2	2	2	1	0	2	0	0	1
Africanomys major	0	0	1	0	0	0	0	2	0	0	0	1	0	1	1	0	0	0	0	0	1	0	?	1	1	2	2	2	1	0	2	0	0	1
Africanomys minor	0	0	1	?	0	0	0	2	0	0	0	-	0	1	1	0	0	0	0	0	1	0	?	1	1	2	2	2	1	0	2	0	0	1
Africanomys cf. solignaci	0	0	1	?	0	0	1	1	0	0	?	?	?	1	1	0	0	0	0	0	1	0	?	0	0	2	2	2	1	0	2	0	0	?

Proafricanomys libanensis nov. gen.	0	0	1	?	0	0	1	1	0	0	?	?	?	1	1	0	0	1	0	0	?	0	?	0	?	2	2	2	1	0	2	0	0	1
Irhoudia robinsoni	1	0	2	?	?	?	?	?	?	?	?	?	?	1	2	1	0	0	1	0	2	0	0	1	2	?	?	?	2	1	2	0	0&1	?
Irhoudia bohlini	1	0	2	?	0	0	0	2	0	0	?	?	?	1	2	1	0	0	1	0	2	0	0	1	2	?	?	?	2	1	2	0	0&1	0
Pellegrinia panormensis	2	1	2	?	-	-	-	-	-	-	-	-	-	?	2	-	1	0	1	1	2	1	0	-	-	-	-	-	2	0	3	0	1	0
Pectinator spekei	1	0	2	0	0	0	0	2	0	0	1	1	0	1	2	1	0	0	0	1	2	0	2	2	2	3	?	?	2	0	3	1	0	0
Ctenodactylus gundi	1	1	2	1	1	0	0	2	0	1	?	?	?	1	2	1	1	0	1	1	2	1	2	2	2	?	?	?	2	0	3	2	1	0
Ctenodactylus vali	1	1	2	1	1	0	0	2	0	1	?	?	?	1	2	1	1	0	1	1	2	1	2	2	2	?	?	?	2	0	3	2	1	0
Massoutiera mzabi	1	1	2	1	1	0	0	2	0	2	-	-	-	2	2	-	1	0	1	1	2	1	1	2	2	-	-	-	2	1	3	0	1	0
Felovia vae	1	1	2	1	?	0	0	2	0	?	?	?	?	1	2	1	1	0	1	1	2	1	0	2	2	?	?	?	2	1	3	0	1	0

References

1. Flynn. L.J. et al. The Siwaliks and Neogene Evolutionary Biology in South Asia. *Fossil Mammals of Asia. Neogene bioestratigraphy and chronology* (eds. Wang, X, Flynn, L. J. & Fortelius, M.) 353–372 (Columbia University Press, New York, 2013).
2. López-Antoñanzas, R. & Knoll, F. A comprehensive phylogeny of the gundis (Ctenodactylinae, Ctenodactylidae). *J. Syst. Palaeontol.* **9**, 379–398 (2011).
3. Rook,L., Abbazzi, L. & Engesser, B. An overview on the Italian Miocene land mammal faunas. *Hominoid Evolution and Climatic Change in Europe, Vol. 1* (eds. Agustí, J., Rook, L. & Andrews, P.) 191-204 (Cambridge University Press, Cambridge, 1999).
4. Wang, B. Y. et al. [Cenozoic stratigraphy in Danghe area, Gansu Province, and uplift of Tibetan plateau.]. *Vert. Palasiatica* **41**, 66–75 (2003). [In Chinese with English abridged version].
5. Wang, X. M. et al. Neogene Mammalian Biostratigraphy and Geochronology of the Tibetan Plateau. Fossil Mammals of Asia. Neogene bioestratigraphy and chronology (eds. Wang, X, Flynn, L. J. & Fortelius, M.) 274–292 (Columbia University Press, New York, 2013).
6. López-Antoñanzas, R., Sen, S. & Sarac, G. A new large ctenodactylid species from the Lower Miocene of Turkey. *J. Vertebr. Paleontol.* **24**, 676–688 (2004).
7. Baskin, J. A. Systematic revision of Ctenodactylidae (Mammalia, Rodentia) from the Miocene of Pakistan. *Palaeovertebrata* **25**, 1–49 (1996).
8. López-Antoñanzas, R. & Sen, S. Systematic revision of Mio-Pliocene Ctenodactylidae (Mammalia, Rodentia) from the Indian subcontinent. *Eclogae Geol. Helv.* **96**, 521–529 (2003).
9. De Bruijn, H., Hussain, S. T. & Leinders, J. M. Fossil rodents from the Murree formation near Banda Daud Shah, Kohat, Pakistan. *Proc. Kon. Nederl. Akad. Wetensch. B* **84**, 71–99 (1981).
10. Sen, S. & Thomas, H. Découverte de rongeurs dans le Miocène moyen de la Formation Hofuf (Province du Hasa, Arabie Saoudite). *C. R. Somm. Soc. Géol. France* **21**, 34–37 (1979).
11. López-Antoñanzas, R. & Sen, S. Ctenodactylids from the Lower and Middle Miocene of Saudi Arabia. *Palaeontology* **47**, 1477–1494 (2004).
12. De Bruijn, H., Boon, E. & Hussain, S. T. Evolutionary trends in Sayimys (Ctenodactylidae, Rodentia) from the Lower Manchar Formation (Sind, Pakistan). *Proc. Kon. Nederl. Akad. Wetensch. B* **92**, 191–214 (1989).
13. López-Antoñanzas, R., Sen, S. & Koufos, G. D. Presence of a ctenodactylid (Rodentia) in the Keramaria Formation (Middle Miocene) of Chios Island (Greece). *Geobios* **38**, 113–126 (2005).

14. Hinton, M. A. C. Diagnoses of new genera and species of rodents from Indian Tertiary deposits. *Ann. Mag. Nat. Hist.* Ser. 10 **12**, 620–622 (1933).

15. Hussain, S. T., Munthe, J., Shah, S. M. I., West, R. M. & Lukacs, J. R. The Daud Khel Local fauna: a preliminary report on a Neogene vertebrate assemblage from the trans-indus Siwaliks, Pakistan. *Milwaukee Pub. Mus. Contr. Biol. Geol.* **16**, 1–16 (1977).

16. Munthe, J. Rodents of the Miocene Daud Khel Local Fauna, Mianwali District, Pakistan. Part 1. Sciuridae, Gliridae, Ctenodactylidae, and Rhyzomyidae. *Milwaukee Pub. Mus. Contr. Biol. Geol.* **34**, 1–36 (1980).

17. Dehm, R., Jacobs, L., Wessels, W., De Bruijn, H. & Hussain, S.T. Fossil rodents from the type area of the Chinji Formation, Siwalik group, Pakistan. *Proc. Kon. Nederl. Akad. Wetensch. B* **85**, 259–263 (1982).

18. Wood, A. E. Fossil rodents from the Siwalik beds of India. *Amer. J. Sci.* **34**, 64–76 (1937).

19. Prasad, K. N. The vertebrate fauna from the Siwalik beds of Haritalyangar, Himachal Pradesh, India. *Palaeont. Indica* **39**, 1–56 (1970).

20. Vasishat, R. N. First record of maxillary dentition of *Sayimys perplexus* (Ctenodactylidae) from the Indian Siwaliks. *Curr. Sci.* **47**, 859–860 (1978).

21. Vasishat, R. N. *Antecedents of early Man in northwestern India: paleontological and paleoecological evidences*. (Inter-India Publications, New Delhi, 1985).

22. Stoetzel, E. Late Cenozoic micromammal biochronology of northwestern Africa. *Palaeogeogr. Palaeoclimatol. Palaeoecol.* 392, 359–381 (2013).

23. Lavocat, R. Le gisement de vertébrés miocènes de Beni Mellal (Maroc): étude systématique de la faune de mammifères. *Notes Mém. Serv. géol. Maroc* **155**, 29–77 (1961).

24. Benammi, M. Nouveaux rongeurs du Miocène continental du Jebel Rhassoul (Moyenne Moulouya, Maroc).*Geobios* **30**, 713–721 (1997).

25. Benammi, M. Nouveaux gisements à rongeurs dans le Miocène moyen continental du Bassin d'Aït Kandoula basin (Maroc). *Geobios* **39**, 589–598 (2006).

26. Jaeger, J. J. Les rongeurs du Miocène moyen et supérieur du Maghreb. *Palaeovertebrata* **8**, 1–166 (1977).

27. Jaeger, J. J. Un cténodactylidé (Mammalia, Rodentia) nouveau, *Irhoudia bohlini* n.g. n. sp. du Pléistocène inférieur du Maroc, rapports avec les formes actuelles et fossiles. *Notes Mem. Serv. Géol. Maroc* **31**, 113–140 (1971).

28. Benammi, M., Calvo, M., Prébot, M. & Jaeger, J. J. Magnetostratigraphy and paleontology of Aït Kandoula Basin (High Atlas, Morocco) and the African-European late Miocene terrestrial fauna exchanges. *Earth Planet. Sci. Lett.* **145**, 15–29 (1996).

29. Heissig, K. Kleinsäuger aus einer obermiozänen (Vallesium) Karstfüllung Ägyptens. *Mitt. Bayer. Staatsslg. Paläont. hist. Geol.* **22**, 97–101 (1982).

30. Wanas, H. A., Pickford, M., Mein, P., Soliman, H. & Segalen, L. Late Miocene karst system at Sheikh Abdallah, between Bahariya and Farafra, Western Desert, Egypt: Implications for palaeoclimate and geomorphology. *Geol. Acta* **7**, 475–487 (2009).
31. Mein, P. & Pickford, M. Vallesian rodents from Sheikh Abdallah, Western Desert, Egypt. *Historical Biol.* **22**, 224–259 (2010).
32. Geraads, D. Rongeurs et insectivores (Mammalia) du Pliocène final de Ahl A1 Oughlam (Casablanca, Maroc). *Geobios* **28**, 99–115 (1985).
33. De Gregorio, A. Intorno a un deposito di roditori e di carnivori sulla vetta di Monte Pellegrino. *Atti Soc Tosc Sci Nat Pisa* **8**, 3–39 (1887).
34. Thaler, L. Les rongeurs (Rodentia et Lagomorpha) du Monte Pellegrino et la question des anciens isthmes de la Sicile. *C. R. Acad. Sci. Sér. D* **274**, 188–190 (1972).
35. Kotsakis, T. Elementi di paleobiogeograpia dei mammiferi Terziari dell' italia. *Hystrix* **1**, 25–68 (1986).
36. Azzaroli, A. Palaeogeography of terrestrial vertebrates in the perityrrhenian area. *Palaeogeogr. Palaeoclimatol. Palaeoecol.* **77**, 83–90 (1990).
37. Villa, P. Early Italy and the colonization of western Europe. *Quat. Int.* **75**, 113–130 (2001).
38. Marra, A. C. Pleistocene mammals of Mediterranean islands. *Quat. Int.* **129**, 5–14 (2005).
39. Marra, A. C. Evolution of Endemic Species, Ecological Interactions and Geographical Changes in an Insular Environment: A Case Study of Quaternary Mammals of Sicily (Italy, EU). *Geosciences* **3**, 114-139 (2013).
40. Ameur, R. Découverte de nouveaux rongeurs dans la formation Miocène de Bou Hanifia (Algérie occidentale). *Geobios* **17**, 167–175 (1984).
41. Robinson, P. & Black, C.C. A small Miocene faunule from near Testour, Beji Gouvernorat, Tunisia. *Ann. Mines Géol.* **26**, 445–449 (1973).
